# Supplementary figures and images for: The PEDtracker: An Automatic Staging Approach for Drosophila melanogaster Larvae
Source: Front Behav Neurosci. 2020 Dec 16;14:612313. doi: 10.3389/fnbeh.2020.612313 (PMC7772430; doi:10.3389/fnbeh.2020.612313)

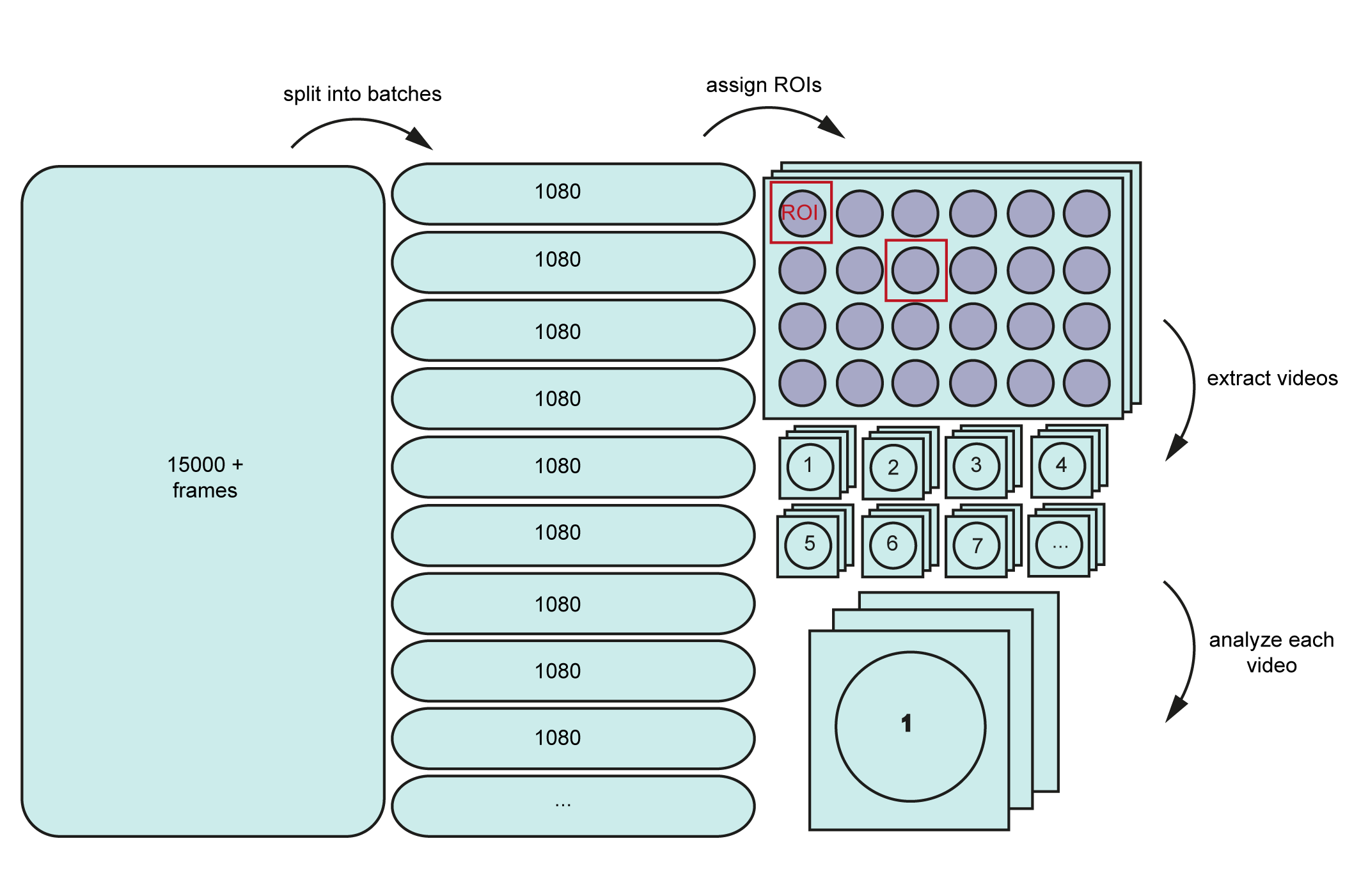

Supplement: Supplementary Figure 1 — Scheme of the Fiji macro script. Frames were distributed in packages of 1080 frames (equivalent to 6 h at 3 fpm) and then cropped in regions of interest (ROIs). ROIs were analyzed with specific parameters. Parameters for analyzed particles were saved in a csv-file. Batches at the border of two developmental stages were analyzed with parameters of both respective stages. [file Image_1.TIF]

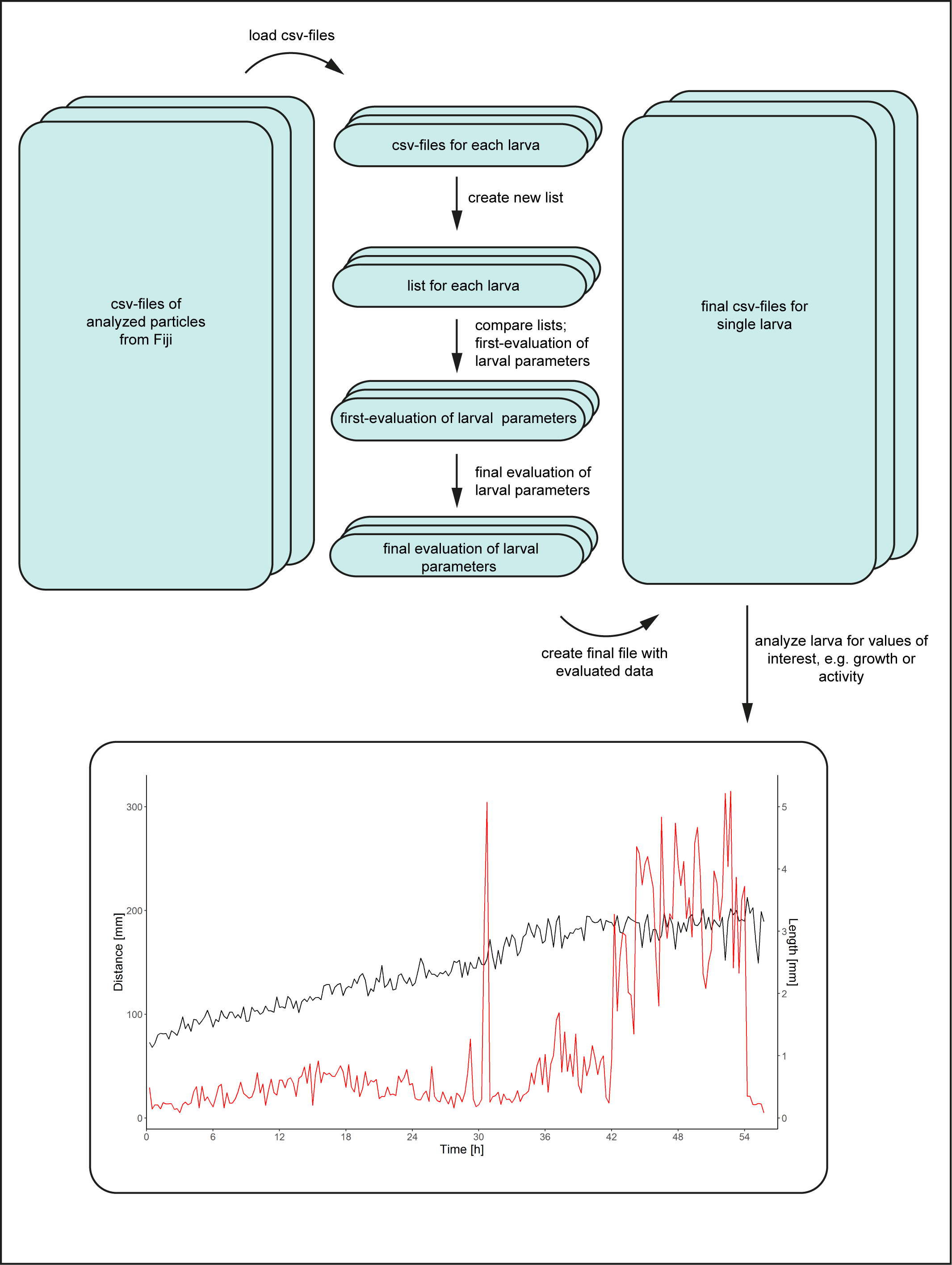

Supplement: Supplementary Figure 2 — Scheme of the R script. Csv-files were loaded in R and combined into a newly created list. Data were evaluated in two steps. First, fitting settings where selected and then objects where analyzed with these settings. After final evaluation of the data, new csv-files were saved and results were plotted using ggplot2. [file Image_2.TIF]

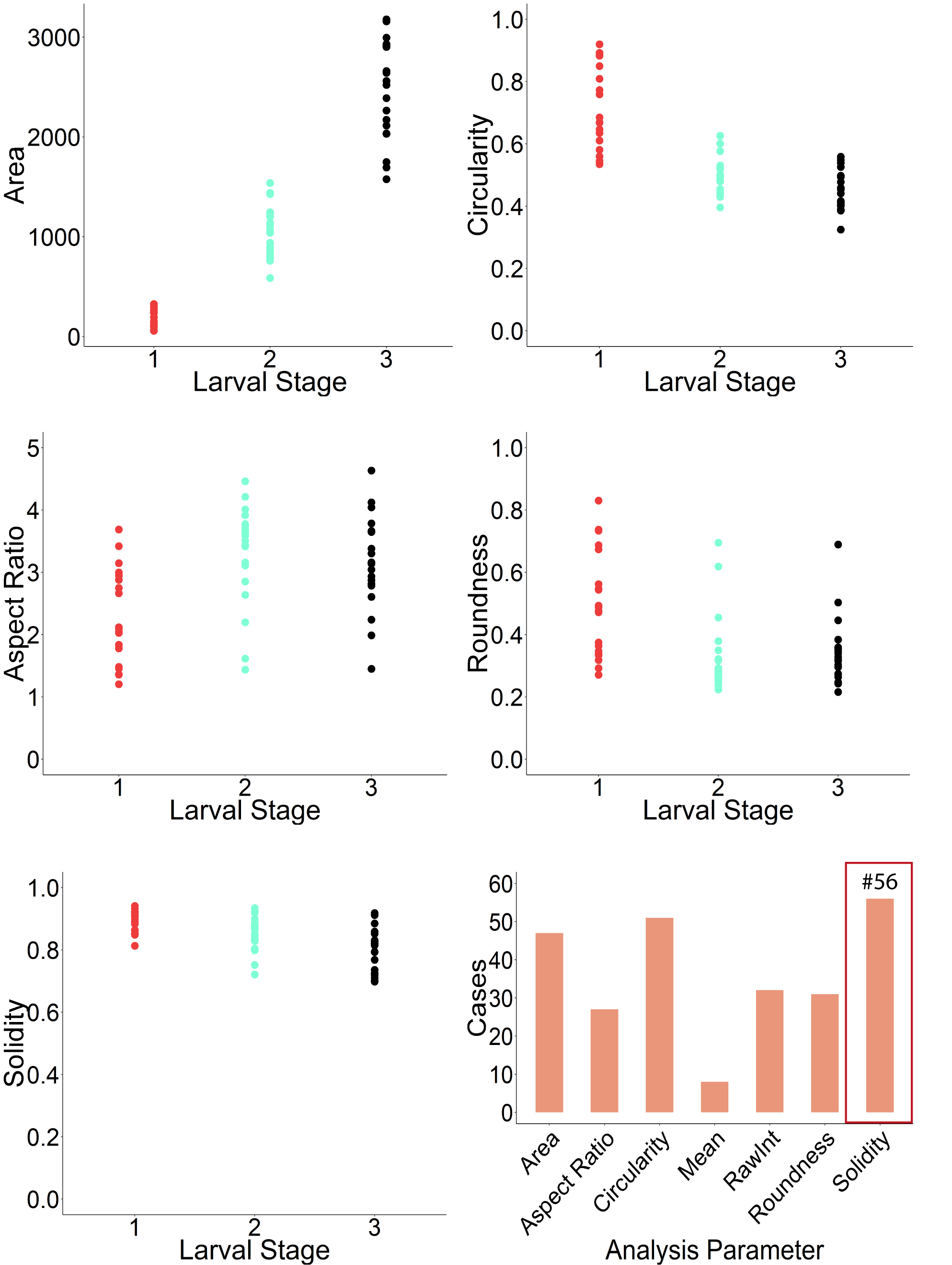

Supplement: Supplementary Figure 3 — Definition of larval parameters for analyzing larval objects in Fiji and R. Parameters (Area, Circularity, Aspect Ratio, Roundness, Solidity) were defined for each larval stage with the objective to reliably sort out non-larval objects. To distinguish one or more objects from a larval object we have examined 60 cases (orange bar plot) for different parameters. In 56 cases (93%) the parameter “solidity” was higher for larval objects. For this reason, we used the value for solidity to distinguish larval from other objects in one frame. [file Image_3.TIF]

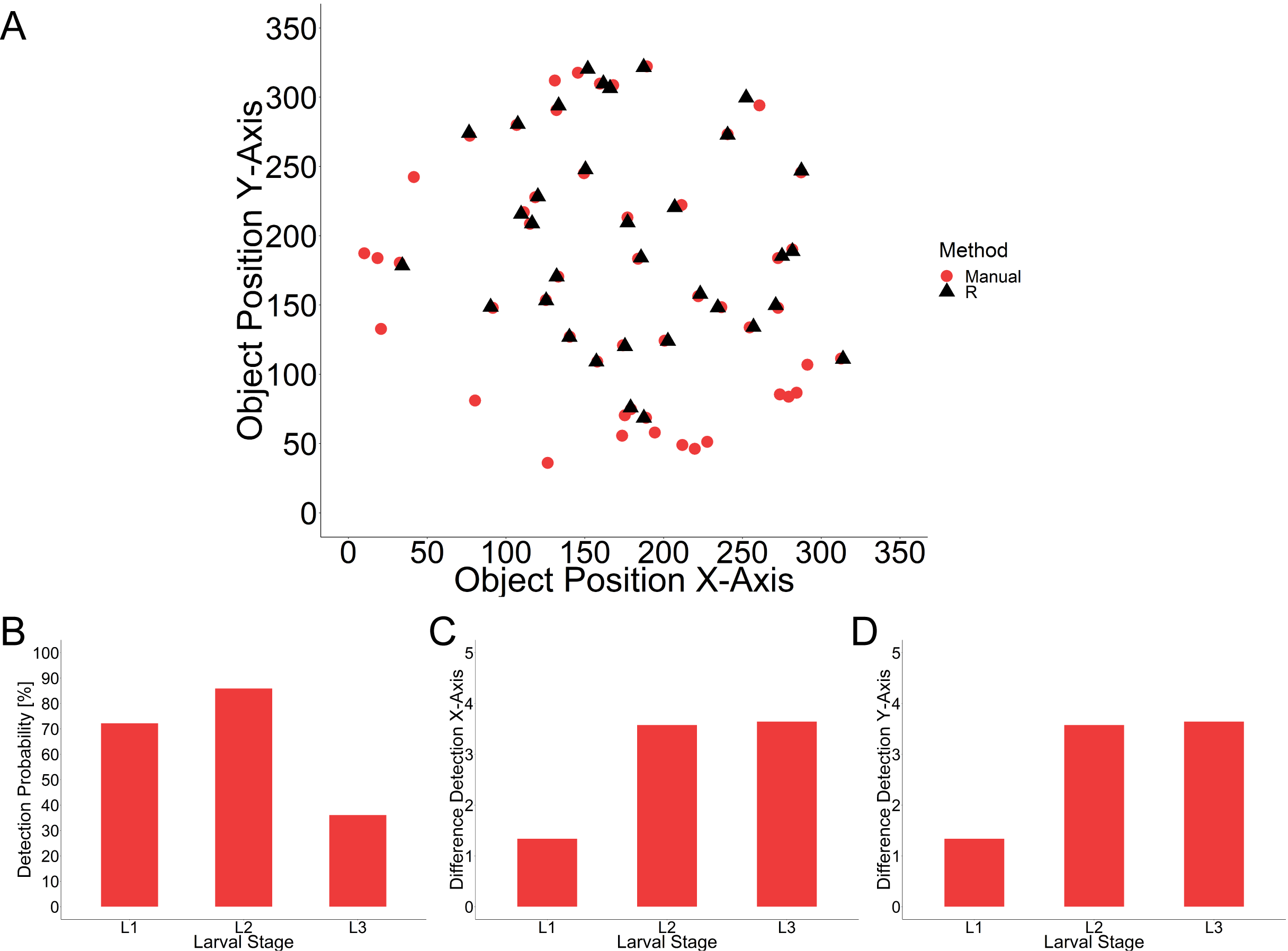

Supplement: Supplementary Figure 4 — Evaluation of the centroid of an object in a frame. (A) Point cloud represents 51 cases of objects. X- and Y-Axis represents the width of the larval bed in pixel. Red dots indicate manually detected objects every 10 frames, black triangles represent the analyzed objects with Fiji and R, respectively. Note that the two-paired points are close together, except for non-detected objects which shows that PEDtracker detects larval objects and avoids false detections. (B) Bar plot indicates the detection probability for larval objects. Note that the detection probability is highest for L2 and lowest for L3. (C) Bar plot indicates the difference of the manual and computational detected object position on the X-axis. The difference of the detection between manual and computational detected object positions on the X-axis is below 5 pixels (lower than one larval length). (D) Bar plot indicates the difference of the manual and computational marked object position at the Y-Axis. The difference of the detection between manual and computational marked object positions on the Y-axis is below 5 pixels (lower than one larval length). [file Image_4.TIF]

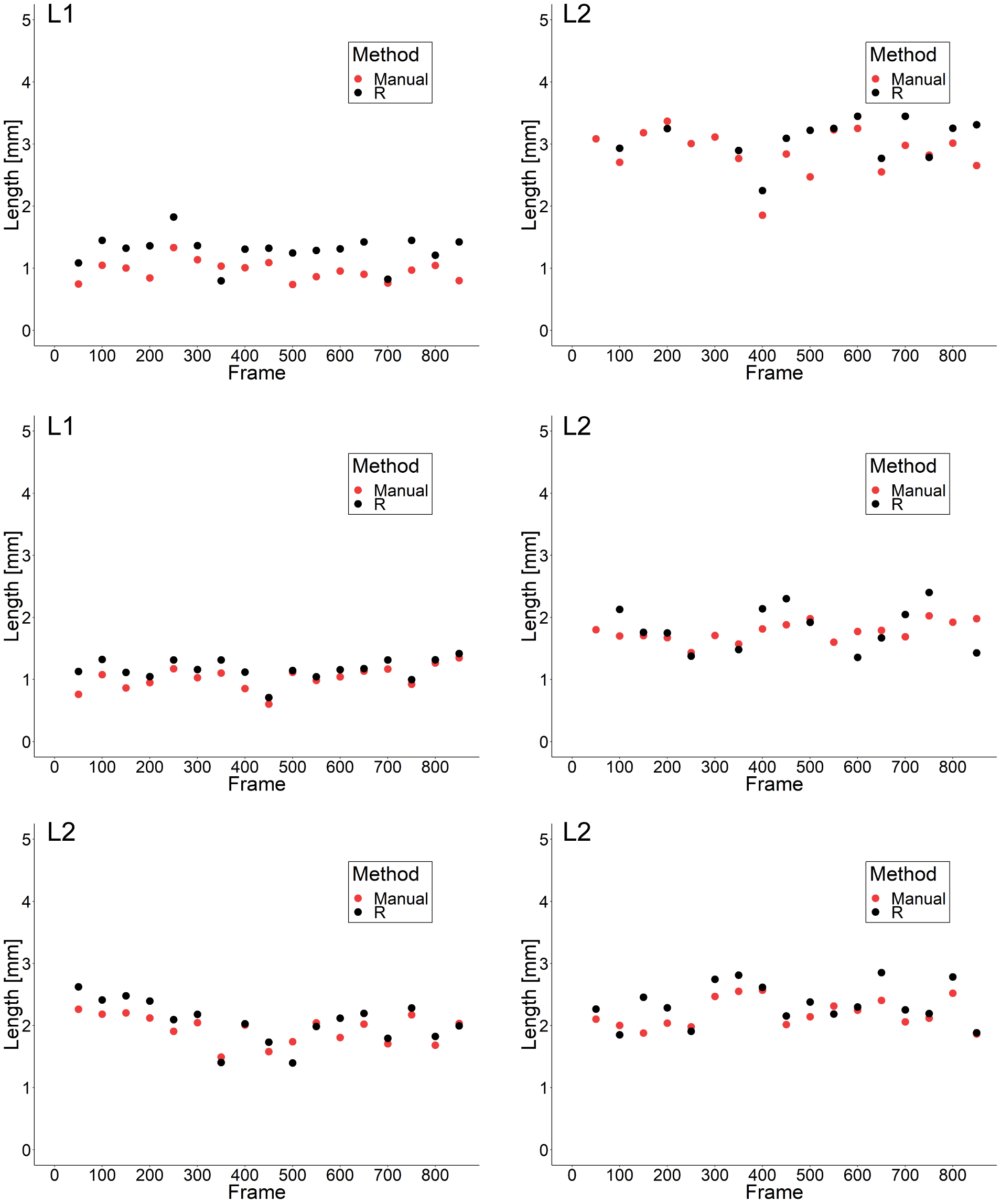

Supplement: Supplementary Figure 5 — Comparison of larval length between manual and computational detection. Note that the lengths of the computational detected larvae are slightly higher than the manual values due to the more precise area determination and resulting longitudinal axis. [file Image_5.TIF]

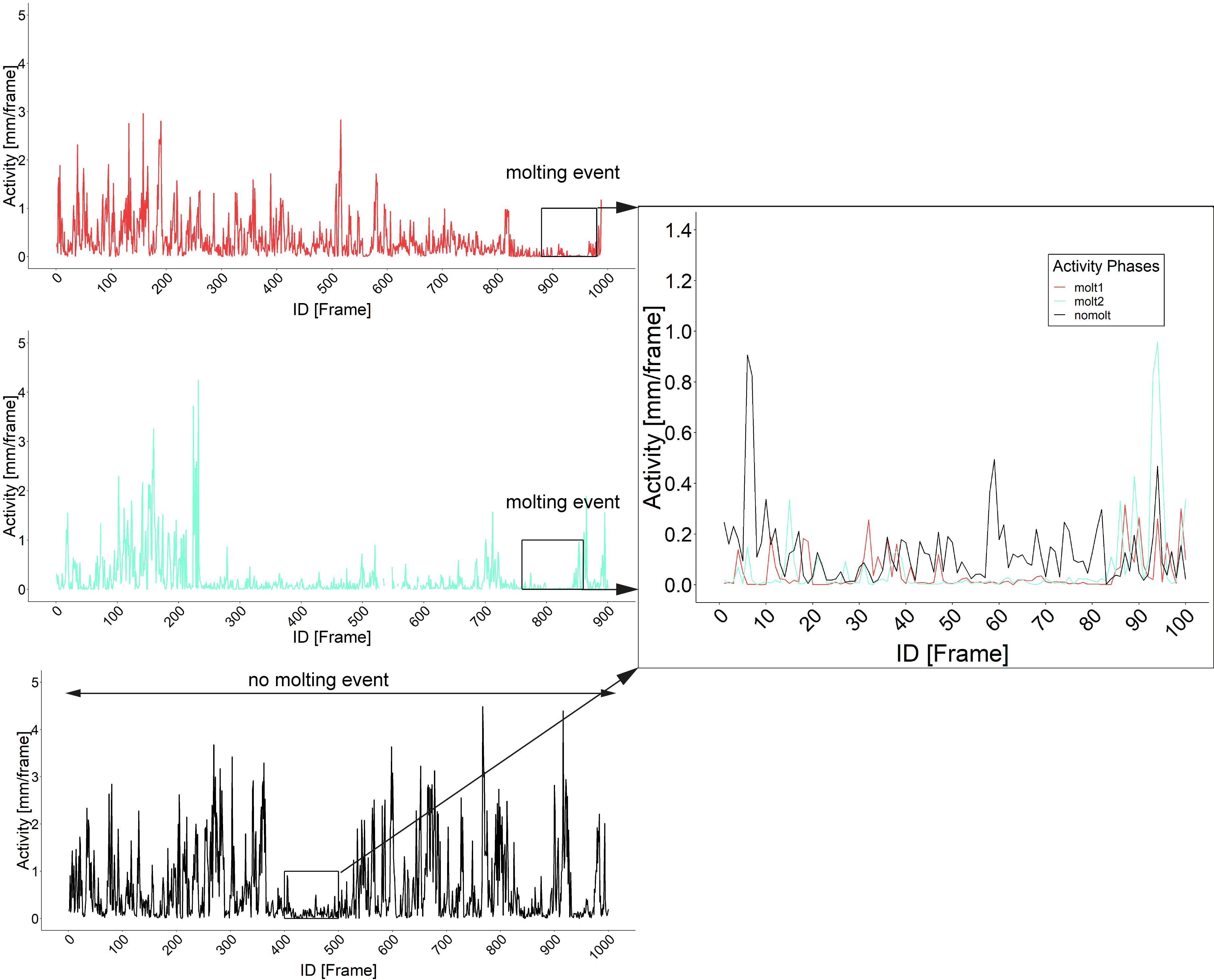

Supplement: Supplementary Figure 6 — Activity pattern from a period of about 1,000 frames (~ 5.5 h) of a molting event and a non-molting event of first instar larvae. Insert compares the level of low activity of a molting event and the non-molting event. Note the phases no activity (light blue and red line). [file Image_6.PNG]

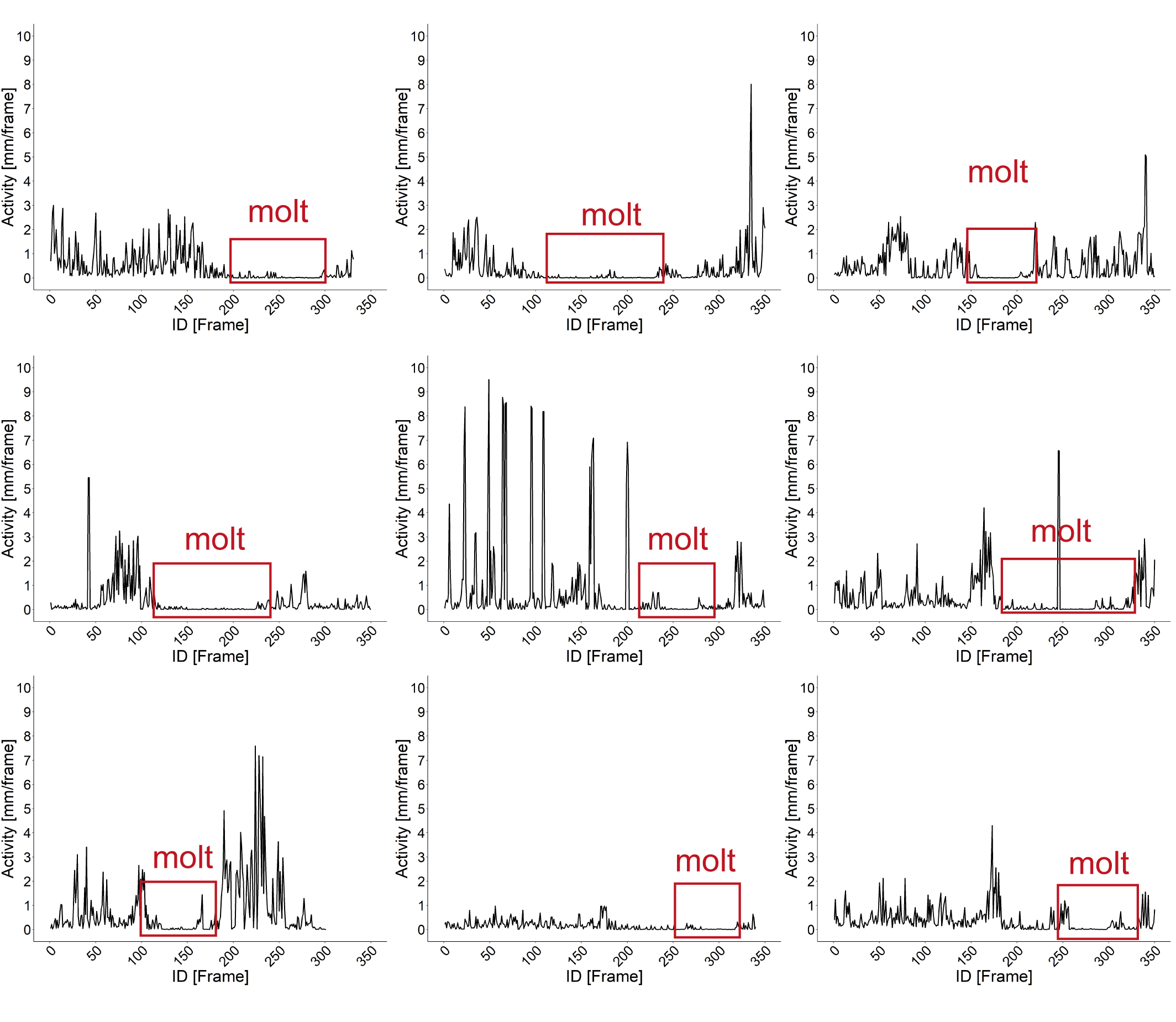

Supplement: Supplementary Figure 7 — Molting events of individual first instar larvae. Note the phases of low activity in all images. Low activity indicates ecdysis behavioral sequence of D. melanogaster larvae. [file Image_7.TIF]

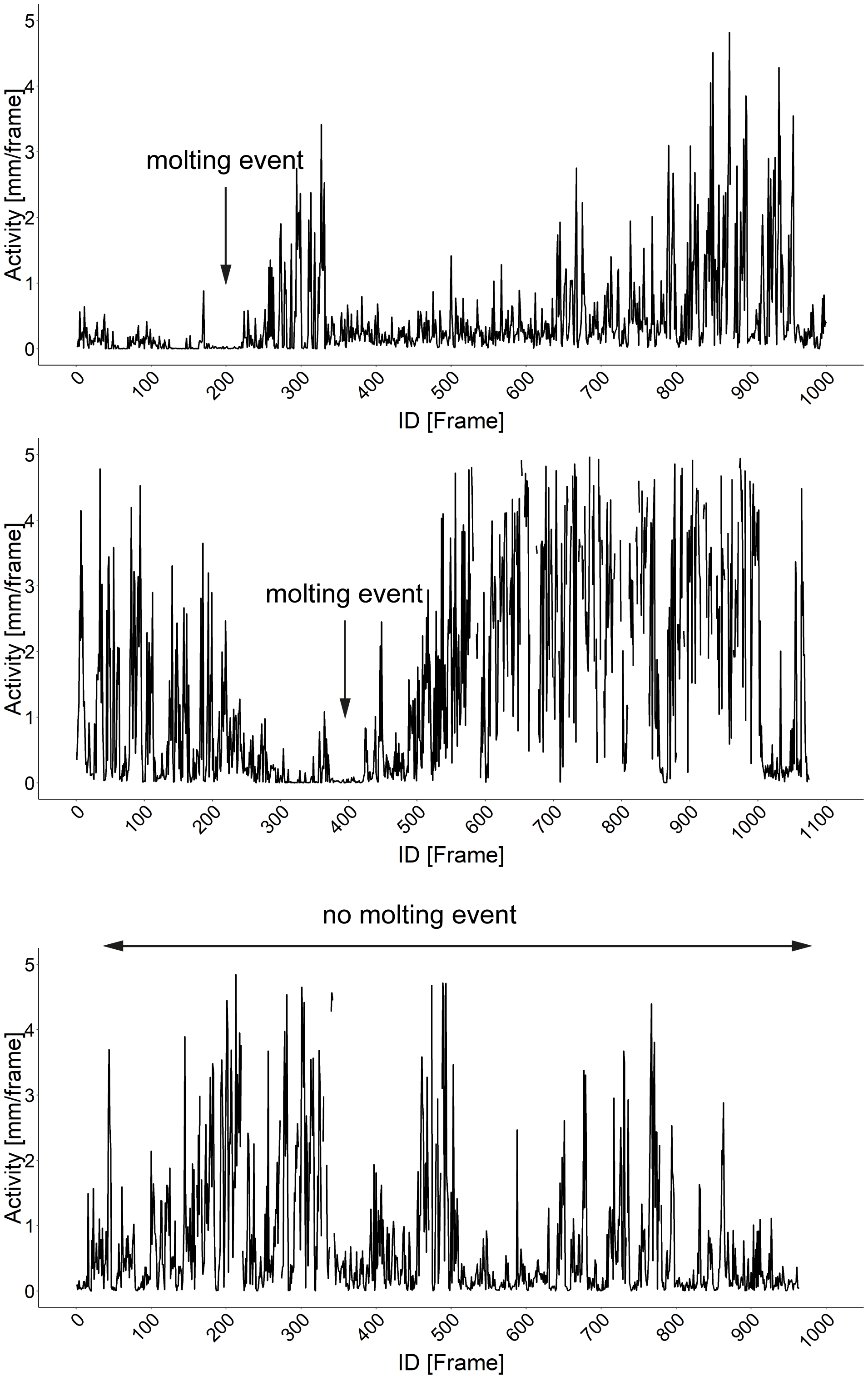

Supplement: Supplementary Figure 8 — Activity pattern from a period of about 1,000 frames (~ 5.5 h) of a molting and a non-molting event of second instar larvae. [file Image_8.TIF]

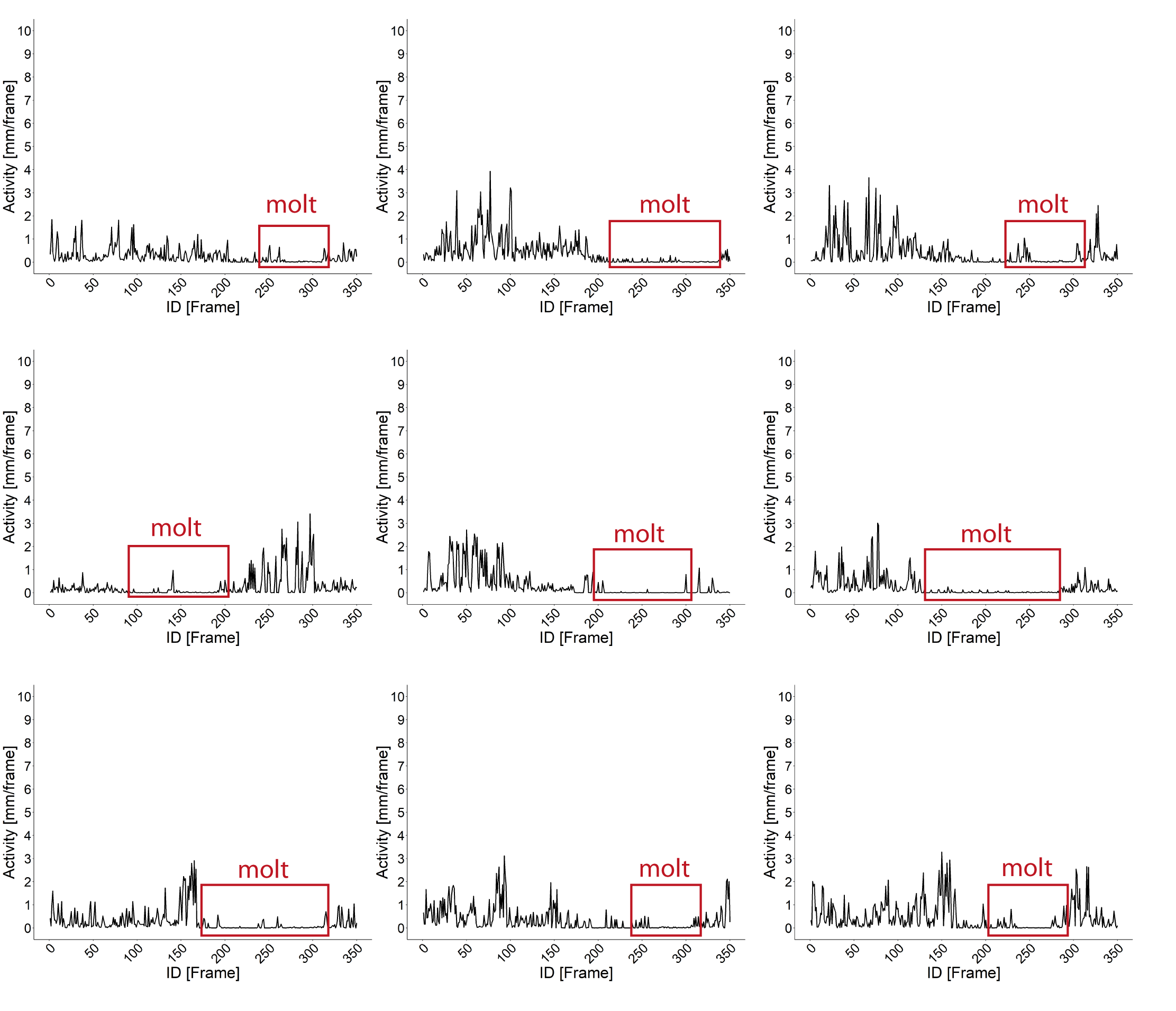

Supplement: Supplementary Figure 9 — Molting events of individual second instar larvae. Note the phases of low activity in all images. Low activity indicates ecdysis behavioral sequence of D. melanogaster larvae. [file Image_9.TIF]
